# Effects of Iron Deficiency on Serum Metabolome, Hepatic Histology, and Function in Neonatal Piglets

**DOI:** 10.3390/ani10081353

**Published:** 2020-08-05

**Authors:** Zhenglin Dong, Dan Wan, Huansheng Yang, Guanya Li, Yiming Zhang, Xihong Zhou, Xin Wu, Yulong Yin

**Affiliations:** 1 Hunan Provincial Key Laboratory of Animal Intestinal Function and Regulation, Hunan international joint Laboratory of Animal Intestinal Ecology and Health, College of Life Sciences, Hunan Normal University, Changsha 410081, Hunan, China; dongzhl@foxmail.com (Z.D.); yhs@hunnu.edu.cn (H.Y.); jerytt@163.com (G.L.); 1347077364@163.com (Y.Z.); 2 Key Laboratory of Agro-Ecological Processess in Subtropical Region, Hunan Research Center of Livestock & Poultry Sciences, South-Central Experimental Station of Animal Nutrition and Feed Science in Ministry of Agriculture, Institute of Subtropical Agriculture, The Chinese Academy of Sciences, Changsha 410125, Hunan, China; xhzhou@isa.ac.cn (X.Z.); wuxin@isa.ac.cn (X.W.)

**Keywords:** neonatal piglets, iron deficiency, metabolomics, metabolite signatures, iron

## Abstract

**Simple Summary:**

Iron deficiency is a serious nutrient deficiency in neonatal pigs during the suckling period in modern intensive farming systems and leads to impaired immune response, infection risks, and retardation of growth. The objective was to determine how iron deficiency in neonatal pigs alters the serum metabolomic profile using quantitative and qualitative analysis by ultra-performance liquid chromatography-tandem mass spectrometer (UPLCMS/MS). The current results revealed that iron deficiency led to a series of metabolic changes involved in tyrosine metabolism, phenylalanine metabolism, bile secretion, primary bile acid biosynthesis, steroid biosynthesis, and upregulated activities of the urea cycle enzymes in the liver of neonatal piglets.

**Abstract:**

Few studies focused on the effects of iron on characterizing alterations of metabolic processes in neonatal piglets. In the present study, 16 neonatal piglets were randomly assigned to two groups. In the first group piglets were given an intramuscularly injection of iron dextran at 150 mg as a positive control (CON) and the second group were not supplemented with iron as a negative control for iron deficiency (ID). At day 8, iron status, serum biochemical parameters, serum metabolome, hepatic histology, and hepatic expression of genes for the metabolism were analyzed. Results indicated that piglets without iron supplementation had significantly reduced iron values and increased blood urea nitrogen concentrations at day 8 (*p* < 0.05). Analysis of serum metabolome revealed that concentrations of serum lysine, leucine, tyrosine, methionine, and cholesterol were significantly decreased while concentrations of 3-Methyldioxyindole, chenodeoxycholate acid, indoleacetic acid, icosadienoic acid, phenylpyruvic acid, pantothenic acid, ursocholic acid, and cholic acid were significantly increased in iron deficient piglets (*p* < 0.05). Furthermore, expressions of cyp7a1 and the urea cycle enzyme (ornithinetranscarbamoylase and argininosuccinate synthetase) were significantly increased in iron deficient pigs (*p* < 0.05). The present experimental results indicated that neonatal piglets without iron supplementation drop to borderline anemia within 8 days after birth. Iron deficiency led to a series of metabolic changes involved in tyrosine metabolism, phenylalanine metabolism, bile secretion, primary bile acid biosynthesis, steroid biosynthesis, and upregulated activities of the urea cycle enzymes in the liver of neonatal piglets, suggesting early effects on metabolic health of neonatal piglets.

## 1. Introduction

Iron is an essential micronutrient for almost all organisms via the catalytic and structural roles playing in a vast range of biological processes including cell proliferation, DNA synthesis, and oxygen transport [1]. Iron is particularly important during the rapid growth and development that occurs following birth due to huge demand in the early postnatal period. As sow’s milk largely provides iron below daily requirements for rapidly growing piglets, neonatal piglets would have to rely on low iron stores in the body at birth and quickly become iron-deficient without an exogenous iron supplementation [2]. Iron deficiency is considered a serious nutritional disorder in suckling piglets in modern intensive farming systems. Iron deficiency is a leading cause of anemia and has been associated with a series of metabolic stresses such as impaired immune response, retardation of growth along with behavioral disorders in pigs [3,4,5]. The underlying iron deficiency remains a concern and it would be of great importance to explore the effects of iron imbalances on characterizing alterations of metabolic processes in suckling piglets.

Metabolomics techniques that rely on the use of analytical platforms, such as nuclear magnetic resonance spectroscopy, gas or liquid chromatography-mass spectrometry (GC-MS/LC-MS) and capillary electrophoresis/mass spectrometry (CE-MS) can be a powerful approach to detect the composition of products and to identify the active metabolites. The metabolic changes directly provide detailed insights into simultaneously interrogating multiple metabolic processes under nutrient deficiencies and diseases, which can allow the discovery of novel biomarkers with potential diagnostic value [6,7]. As a useful metabolomic method with high sensitivity, peak resolution, and reproducibility, we hypothesized LC/MS-based untargeted metabolomic analysis can provide valuable information for assessing iron deficiency anemia. Yet, few studies focused on characterizing alterations of metabolic processes in iron deficiency status in neonatal piglets. In the current study, the newborn piglets were either supplemented with iron or not, and the impact of iron deficiency on the overall body metabolic profiles (the metabolome) was performed through quantitative and qualitative analysis by ultra-performance liquid chromatography-tandem mass spectrometer (UPLCMS/MS). By this approach, it was possible to classify the samples on the basis of their different metabolism for studying its metabolic consequences.

## 2. Materials and Methods

### 2.1. Ethics Statement

The animal experiments were conducted according to the animal welfare requirements and approved by the Animal Protocol Review Committee of the Subtropical Agriculture, Chinese Academy of Science (No. ISA-2017-012).

### 2.2. Animals and Proceeding

A total of 16 one-day-old male neonatal piglets (Large White × Landrace × Duroc, 1.50 ± 0.05 kg) were selected and randomly allocated into two groups. In the first group piglets were given an intramuscular injection of iron dextran at 150 mg as a positive control (CON) and the second group were not supplemented with iron as a negative control for iron deficiency (ID). During the experiment period, all piglets were mixed and raised by mother sows (eight piglets per litter with four from each treatment) and given free access to milk in farrowing crates with conditions of approximately 70% humidity and a temperature of 30 ± 2 °C. 

### 2.3. Sample Collection

On day 8, all the piglets were weighed and euthanized with prior anesthesia. The sodium pentobarbital solution (Merck, Darmstadt, Germany) was injected and the piglets were killed by jugular puncture. Whole blood was collected with EDTA-K2 anticoagulant tubes (Aosaite Medical Devices Co., Ltd., Shangdong, China) from the piglets by jugular venepuncture and submitted for hematology analysis immediately. Serum samples were obtained from blood samples after centrifugation and stored at −80 ℃ until analysis. Liver tissues were weighed, dissected, and were sampled (0.5 × 0.5 cm) at about 20 cm and fixed in formaldehyde for histologic evaluation or snap frozen in liquid nitrogen and stored at −80 °C until further analysis. 

### 2.4. Blood Parameters

Indexes such as red blood cells (RBC) count, hemoglobin (Hb), hematocrit (HCT), mean cell volume (MCV), mean corpuscular hemoglobin (MCH), and mean corpuscular hemoglobin concentration (MCHC) were determined using a Blood Cell Analyzer on a Roche Hitachi 917 Chemistry Analyzer. Biochemical parameters such as aspartate transaminase (AST), alanine transaminase (ALT), alkaline phosphatase (ALP), blood urea nitrogen (BUN), glucose, total proteins (TP), albumin, serum iron, and ferritin were measured in the serum samples using an automated chemistry analyzer (Olympus AU 400, Beckman Coulter/Olympus).

### 2.5. Ultra-High Performance Liquid Chromatography (UHPLC) Combined with MASS Spectrometry (MS)

Collected serum samples were acquired by the LC-MS system followed machine orders. Samples were isolated, treated, and injected into the HPLC-MS according to the method [8]. All chromatographic separations were performed using an ultra-high-performance liquid chromatography (UPLC) system (Waters, Milford, UK). An ACQUITY UPLC BEH C18 column (100 × 2.1 mm, 1.7 μm, Waters, Milford, UK) was used for the reversed-phase separation. A high-resolution tandem mass spectrometer Xevo G2 XS QTOF (Waters, Milford, UK) was used to detect metabolites eluted from the column.

An approach based on the periodic analysis of a quality control sample (QC sample) together with the true samples is accepted to ensure that data are of comparable high quality within an analytical run in metabolic profiling [9]. The raw data were converted into CDF format using Masslynx version 4.1 (Waters Corp., Manchester, UK) and imported into Progenesis QI software (version 2.2) for data alignment, normalization, and peak picking. After the generation of a data matrix with retention time (RT), mass-to-charge ratio (m/z) values and peak intensity, multivariate statistical analysis using principal component analysis (PCA) and partial least-squares discriminant analysis (PLS-DA) was used to validate metabolites at a univariate level using FDR (false discovery rate) test with the critical *p*-value set to not higher than 0.05. Candidate metabolites were identified by searching the exact molecular mass data from redundant m/z peaks against the online HMDB (http://www.hmdb.ca/) database and were confirmed by comparison of their MS/MS spectra and retention time with characteristic ions and fragmentation patterns of the compound. The differentially abundant metabolites were cross-referenced to pathways by further searching commercial databases, including KEGG (http://www.genome.jp/kegg/) and MetaboAnalyst.

### 2.6. Liver Index and Histological Analysis

Liver weights were analyzed on an absolute basis, as well as a proportion relative to individual pig body weights. The samples of liver tissue fixed in formaldehyde were embedded in paraffin, sliced into 5-μm thickness. Hematoxylin and eosin (HE) staining was performed and analyzed by microscopy according to the scoring system [10]. Photomicrographs were acquired with magnifications using an Olympus BX51 microscope (Olympus Optical Company, Shanghai, China).

### 2.7. Reverse Transcription and Quantitative Real-Time PCR

The liver samples were pulverized under liquid nitrogen and total RNA was isolated using TRIzol reagent (Invitrogen, Breda, Netherlands). After DNase I treatment, the RNA was cleaned up by the RNeasy Kit (Qiagen, Hilden, Germany) and then quantitated by NanoDrop ND-1000 spectrophotometer (NanoDrop Tech/Thermo, Waltham, USA). Then, RNA integrity was assessed using standard denaturing agarose gel electrophoresis. Quantitative analysis of PCR was carried out triplicate on a Lightcycler 480II system (Roche, Basel, Switzerland) using a SYBR Premix Ex Taq (Thermo, Waltham, USA). The PCR reaction consisted of 5.0 μL SYBR Premix Ex Taq, 0.5 μL cDNA, 3.7 μL RNase free water, and 0.4 μL forward/reverse primer (10 mmol/L) in a total volume of 10 μL. Cycling conditions were 95 °C 10 min, followed by 45 cycles of 95 °C 30 s, 60 °C 30 s, and 72 °C 30 s. Real-time PCR primer sequences for selected genes (Table S1) were designed and synthesized by Sangon Biotech (Shanghai, China). The cycle threshold value was analyzed using Lightcycler 480II system (Roche, Basel, Switzerland) and fold differences in expression concentrations were calculated using the 2^−ΔΔCt^ method, with GAPDH as housekeeping gene to normalize target gene transcript concentrations.

### 2.8. Statistical Analysis

Data were presented as mean with SEM. All statistical analyses were performed using the SPSS 17.0 (SPSS INC., Chicago, IL, USA). The significance of the difference between two groups was analyzed using Student’s unpaired *t*-test followed by Dunnett’s test (two-sided) for the hypothesis of equal means. Probability values <0.05 were considered statistically significant. The acquired LC-MS data was analyzed by PCA and PLS-DA using multivariate analysis. The corresponding variable importance of projection (VIP) values greater than 1, FDR <0.05, and Fold Change >1.2 or <0.8 were selected as differential metabolites.

## 3. Results 

### 3.1. Growth Performance

Performance results on body weight are presented in Table 1. On day 8, there were no significant differences in body weight (*p* > 0.05). 

### 3.2. Iron Status

Iron profile parameters are presented in Table 2. For the hematological analyses, Hb, HCT, MCH, and MCV values were significantly decreased in ID groups (*p* < 0.05). Furthermore, concentrations of total iron and ferritin were significantly decreased in piglets without iron supplementation on day 8 (*p* < 0.05). 

### 3.3. Serum Biochemical Indices

Serum biochemical indices are presented in Table 3. At day 8, serum BUN concentration was significantly greater in piglets without iron supplementation (*p* < 0.05). No significant differences in TP, ALB, GLU, TG, ALT, AST, and ALP were observed between the two groups (*p* > 0.05). 

### 3.4. Analysis of Serum Metabolomics

**Figure 1:**
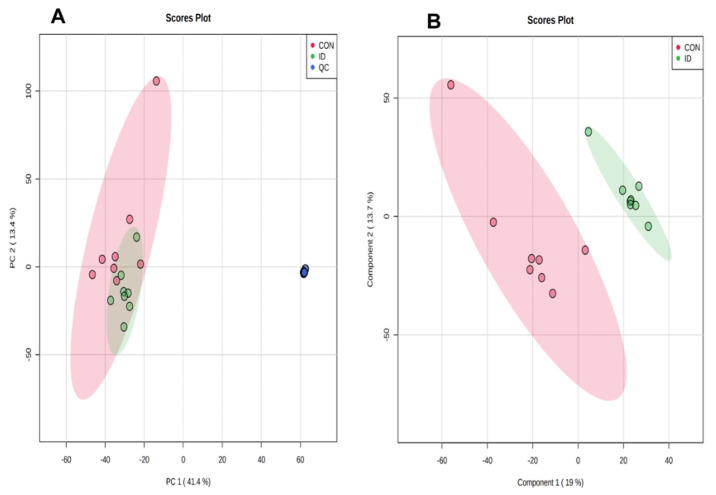
(**A**) Principal component analysis (PCA) score plot of serum metabolites of neonatal piglets with or without iron supplementation. (**B**) Partial least squares discriminant analysis (PLS-DA) score plot of metabolites of pigs with or without iron supplementation.

As shown in Figure 1, the principal component analysis (PCA) scores plot representation of QC samples clustered together. Thus, the metabolic features demonstrated acceptable reproducibility and stability. PLS-DA revealed a clear and statistically significant separation between ID groups and normal controls, which demonstrated that diversity of serum metabolite concentrations existed between these two groups. With the LC-MS method, 221 metabolites were obtained. A total of 19 metabolites were identified significantly different. 

As shown in Table 4, our study showed that concentrations of serum lysine, leucine, tyrosine, methionine, and cholesterol were significantly decreased while concentrations of 3-Methyldioxyindole, chenodeoxycholate acid, indoleacetic acid, icosadienoic acid, phenylpyruvic acid, pantothenic acid, ursocholic acid, and cholic acid were significantly increased in iron deficient piglets (*p* < 0.05). 

**Figure 2:**
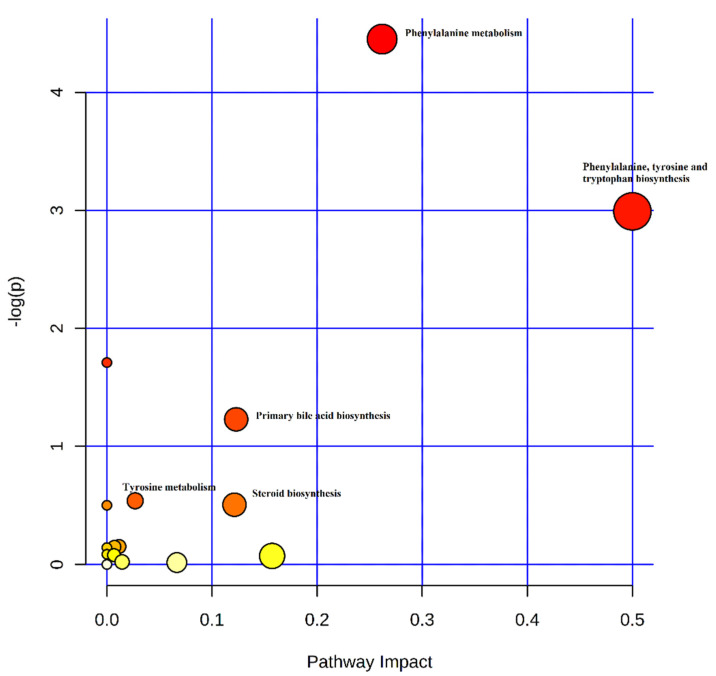
Enriched pathways of serum metabolites in neonatal piglets affected by iron deficiency. Each node marks a pathway, with larger sizes and darker colors representing higher pathway enrichment and higher pathway impact values.

Analysis of the specific metabolic pathways using KEGG (www.genome.jp/kegg/) pathway enrichment analysis indicated that altered metabolites play important roles in steroidogenesis, tyrosine metabolism, tryptophan metabolism, phenylalanine metabolism, bile secretion, and primary bile acid biosynthesis (Figure 2). 

### 3.5. Liver Index and Histological Analysis

**Figure 3:**
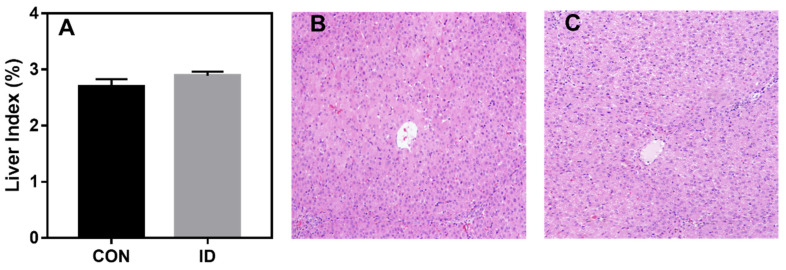
(**A**) Liver index and histological analysis of (**B**) neonatal piglets with or (**C**) without iron supplementation (original magnification, ×200).

Liver index or morphology is depicted in Figure 3. No significant differences in liver index (*p* > 0.05) and morphology were observed in piglets between two groups.

### 3.6. mRNA Expression of Key Genes Related to Hepatic Metabolism

**Figure 4:**
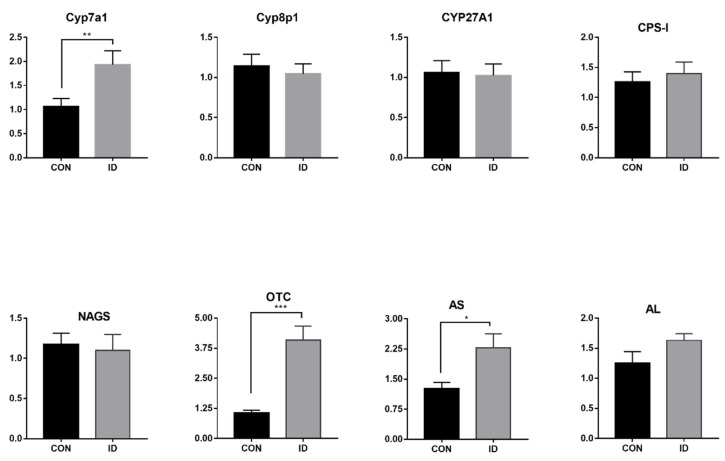
mRNA expression of genes related to the enzymes of hepatic bile acids synthesis and urea cycle in piglets with or without iron supplementation. Cyp7a1, cholesterol 7α-hydroylase; Cyp8p1, Sterol 12α-hydroxylase; CYP27A1, sterol 27 hydroxylase; CPS-I, carbamyl phosphate synthetase I; OTC, ornithinetranscarbamoylase; NAGS, N-acetylglutamate synthase; AS, argininosuccinate synthetase; AL, argininosuccinase lyase. * indicates statistical significance, *p* < 0.01, ** indicates statistical significance, *p* < 0.05, *** indicates statistical significance, *p* < 0.001.

Expression of genes related to enzymes of bile acid synthesis (Cyp7a1, Cyp8p1, and CYP27A1) and urea cycle are presented in Figure 4. Iron deficiency increased the hepatic mRNA expression of cyp7a1, OTC, and AS (*p* < 0.05). The expression of CYP27A1 and did not differ between two groups (*p* > 0.05). There were also no significant differences in the mRNA expression of N-acetylglutamate synthase, carbamoyl-phosphate synthase, and argininosuccinate in liver tissue (*p* > 0.05) of piglets supplemented with iron or not.

## 4. Discussion

In the phase of rapid neonatal growth, neonatal pigs just rely on low iron stores in the body at birth and low iron content in the colostrum/milk [11]. Supplementation of iron via intramuscular injection is needed, otherwise they easily become iron deficient. Iron is one of the raw materials for the synthesis of hemoglobin, and suckling piglets must retain 21 mg of Fe/kg of BW gain to maintain satisfactory hemoglobin [12]. In our study, Hb concentrations in piglets without iron supplementation were decreased to 74.3g/L on day 8, which is far below normal hemoglobin concentrations of 100 g/L. HCT, MCH, and MCV values that are very sensitive indicators of iron deficiency in this group were also significantly decreased. Furthermore, typical markers for measuring available iron, such as serum iron and iron storage protein ferritin, were significantly decreased. A previous study observed a decrease in Hb concentration and HCT percentage at 10 days of age in pigs receiving no supplemental iron [13]. Other reported that a decrease in these clinical biochemical parameters indicated gradually developing iron deficiency in newborn pigs [14]. We provide evidence that the piglets without iron supplementation drop to borderline anemia within 8 days after birth. 

Changes in iron availability and distribution have significant effects on numerous biological processes. Previous studies have indicated iron overload could induce metabolites changes in the blood such as ornithine, citrulline, arginine, malic acid, aspartic acid, lactic acid, and cysteine [15]. Our results show that iron deficiency causes significant dynamic changes in the metabolite balance of the serum of neonatal piglets. Metabolites identified in our study mainly belong to organic acids and derivatives, benzenoids, lipids, and lipid-like molecules, indoles, and derivatives, etc. We found significantly reduced concentrations of lysine, leucine, and methionine in the serum of iron deficient piglets. It is known that some of the enzymes of amino acid biosynthesis require iron in the form of iron-sulfur clusters. It is reported that homoaconitase contains iron-sulfur clusters as cofactors and converts homocitrate to homoisocitrate in the lysine biosynthetic pathway. Isopropylmalate dehydrogenase (Leu1), an Fe-S cluster protein which is required for leucine biosynthesis, is regulated by iron availability [16]. While iron-dependent enzymes were altered, changes of amino acid biosynthesis may occur. Furthermore, an increased concentration of serum indole-3-carbaldehyde and indoleacetic acid were observed in the serum of iron deficient pigs. These compounds play critical roles in tryptophan metabolism. Indoleacetic acid is a breakdown product of tryptophan metabolism. Associations between tryptophan and iron metabolism have been observed in individuals with and without iron deficiency [17]. Enhanced tryptophan degradation by indoleamine 2, 3-dioxygenase activity is considered to be involved in the drop of blood concentrations of hemoglobin and the development of anemia [18]. According to our results, iron deficiency had a significant impact on the tryptophan metabolism of neonatal piglets.

We also found that the concentrations of tyrosine, hippuric acid, and 3-Methoxytyramine was significantly decreased while benzoic acid and phenylpyruvic acid values were significantly decreased in iron deficient pigs. The different activity changes of these metabolites are associated with phenylalanine metabolism and tyrosine metabolism. Phenylpyruvic acid belongs to keto-acid that is an intermediate or catabolic byproduct of phenylalanine metabolism. Tyrosine and phenylalanine are precursors for the catecholamine neurotransmitters dopamine, norepinephrine, and epinephrine, which are associated with brain functions [19]. Low concentrations of these amino acid profiles in the serum may reduce the conversion of dopamine. Iron is found be a cofactor for some enzymes including tyrosine hydroxylase and phenylalanine hydroxylase involved in the synthesis of dopamine [20]. Iron deficiency has widespread short and long-term effects on dopamine metabolism due to the dependence of the neurotransmitter on these iron-containing enzymes [21,22,23]. Our study indicated that iron deficiency anemia negatively affected precursors of neurotransmitters, and may subsequently perturb neural development of suckling piglets.

The metabolites associated with cholesterol metabolism and bile secretion and primary bile acid biosynthesis were detected. We observed decreased cholesterol and 6-Mercaptopurine, increased cholic acid, ursocholic acid, and chenodeoxycholic acid in iron deficient piglets. Impaired fatty acid synthesis and lipid metabolism were noted in iron-deficient humans [24,25]. In the rat study, iron deficiency anemia tends to decrease serum cholesterol concentrations [26,27]. Furthermore, we found iron deficiency may have a significant impact on the regulation of bile acid synthesis and excretion, thus on bile formation. A previous study in rats indicated that iron deficiency substantially induced the transcription of cholesterol transporters and bile acids synthetic enzymes, therefore decreases plasma concentrations of cholesterol and increases biliary secretion of cholesterol [28]. Disrupted bile acid production has also been described in iron-deficient anemic rats and monkeys [29,30]. In the metabonomics analysis, we detected increased cholic acid in response to iron deficiency. It is reported that higher concentrations of bile acids in iron deficiency could indicate a compensatory response to maximize intestinal absorption of iron in an acidic milieu [31]. In the current study, the liver index and morphology were not changed. Such results revealed that short-term anemia would not lead to histological changes in suckling piglets. However, significantly increased cyp7a1 expression was observed in the liver of iron deficient piglets. Bile acids (or bile salts) formed from cholesterol are primarily synthesized in the liver from chenodeoxycholic acid and cholic acid. Cyp7a1 initiated catalyzed sterol side chain oxidation to formation of a bile acid. According to the current study, bile acid synthesis was induced in iron deficient pigs. 

In our study, an elevation in BUN values was observed in response to an inadequate iron status. The serum BUN concentrations are useful indicators of protein metabolism status and amino acid utilization in vivo. It can be used to quantify N utilization and excretion rates. The liver not only tightly regulates iron homeostasis by producing an iron regulatory hormone called hepcidin, but also is the central site for nitrogen metabolism and urea synthesis in the body. Animal data demonstrated significant differences in urinary metabolic profiles between iron overload rats and their control rats, which revealed a connection of urea cycle with iron metabolism in the liver [32]. Other reports indicated hepcidin correlated significantly with urea [33]. Very little information is available about the changes in nitrogen metabolism occurring under iron deficiency. Thus, we checked if the iron depletion may also modify urea cycle metabolism. We further evaluated the enzyme mRNAs of these enzymes (N-acetylglutamate synthase, carbamoyl-phosphate synthase, ornithine carbamoyltransferase, argininosuccinate synthetase, and arginase) in liver tissue. N-acetylglutamate synthase (NAGS) activates carbamoyl phosphate synthetase-I (CPS-I) to start urea. OTC catalyzes the carbamylphosphate and ornithine to citrulline. In the current study, the result showed the hepatic mRNA expression of OTC and AS were induced in the iron deficiency group. A previous study reported that the upregulation of argininosuccinate lyase during iron-deficient anemia may apply nitrogen atoms for the urea cycle in a DNA microarray study [34]. Similar to this, we found that iron deficiency induced activities of the urea cycle. As a result, enhanced concentrations of urea in blood was observed in iron deficient pigs.

## 5. Conclusions

The present experimental results indicated neonatal piglets without iron supplementation drop to borderline anemia within 8 days after birth. LC-MS analysis of serum provided a sensitive platform to interrogate the metabolomic profile associated with early iron deficiency of neonatal piglets. We found iron deficiency led to a series of metabolic changes involved in steroidogenesis, tyrosine metabolism, tryptophan metabolism, phenylalanine metabolism, bile secretion, primary bile acid biosynthesis, and upregulated activities of the urea cycle enzymes in neonatal piglets. According to our study, investigation of metabolic signatures provided a novel and useful tool for getting deeper insights into metabolic reaction and for revealing potential biomarkers of iron deficiency.

## Figures and Tables

**Table 1:** Growth performance of neonatal piglets with or without iron supplementation.

Items	CON	ID	SEM	*p*-Value
Day 1 (kg)	1.51	1.49	0.68	0.9555
Day 8 (kg)	2.24	2.10	0.98	0.2961
Gain (kg/d)	0.74	0.60	0.53	0.5647

CON = piglets were given iron administration; ID = piglets were not given iron administration; *p*-Value < 0.05 indicates significant difference. Values are expressed as means with Standard Error of Mean (SEM).

**Table 2:** Iron status biomarkers of neonatal piglets with or without iron supplementation.

Items	CON	ID	SEM	*p*-Value
RBC (× 10^12^)	4.47	4.04	0.36	0.2781
HCT (%)	30.97	23.44	1.64	0.007
MCH (pg)	21.56	17.62	0.6	0.003
MCHC (g/L)	315.83	307.8	4.16	0.4541
MCV	68.62	58.23	1.74	0.0013
Hb (g/L)	100.29	74.29	6.88	<0.001
Serum iron (ug/dL)	34.82	24.1	2.04	0.0104
Ferritin (ug/L)	1.98	1.25	0.104	0.0350

CON = piglets were given iron administration; ID = piglets were not given iron administration; *p*-Value < 0.05 indicates significant difference. Values are expressed as means with Standard Error of Mean (SEM). RBC = red blood cell count, HCT = hematocrit, MCH = mean corpuscular hemoglobin, MCHC = mean corpuscular hemoglobin concentration, MCV = mean corpuscular volume, Hb = hemoglobin.

**Table 3:** Serum biochemical parameters of neonatal piglets with or without iron supplementation.

Items	CON	ID	SEM	*p*-Value
TP (g/L)	55.24	58.85	1.65	0.086
BUN (mmol/L)	2.54	6.27	1.02	0.005
ALB (g/L)	20.03	21.4	0.79	0.207
GLU (g/L)	5.25	5.6	0.28	0.278
TG (g/L)	0.65	0.84	0.16	0.246
ALT (U/L)	51.2	41.8	4.05	0.711
AST (U/L)	51.1	54.6	3.56	0.646
ALP (U/L)	876.6	686.7	52.93	0.113

CON = piglets were given iron administration; ID = piglets were not given iron administration; *p*-Value < 0.05 indicates significant difference. Values are expressed as means with Standard Error of Mean (SEM). AST = aspartate transaminase, ALT = alanine transaminase, ALP = alkaline phosphatase, BUN = blood urea nitrogen, TP = total proteins, ALB = albumin, GLU = glucose, TG = triglyceride.

**Table 4:** Significantly altered metabolites identified in the serum of neonatal piglets with or without iron supplementation.

Mass-to-Charge Ratio (m/z)	Metabolites	FC	Adjusted *p*-Value	VIP
6.74_183.0807m/z	Methionine	0.21852	1.23E-07	5.612417
6.74_105.0336m/z	Benzoic acid	0.248168	6.61E-07	5.321288
0.51_169.0947m/z	Lysine	0.593345	0.000219	1.824616
8.86_369.3518m/z	Cholesterol	0.460344	0.003186	2.092795
0.63_132.1021m/z	Leucine	0.753322	0.003469	1.336155
8.62_326.3051m/z	Icosadienoic acid	1.60629	0.006241	1.561216
4.47_147.0441m/z	Phenylpyruvic acid	2.824423	0.012168	4.394213
4.89_146.0603m/z	Indole-3-carbaldehyde	1.976067	0.013175	3.198039
4.47_176.0705m/z	Indoleacetic acid	1.851496	0.013453	2.32749
11.07_423.3227m/z	Calcidiol	1.358637	0.015394	1.42136
5.53_190.0854m/z	3-Methoxytyramine	0.510584	0.015693	2.803578
3.98_162.0547m/z	Hippuric acid	2.003734	0.017542	3.201292
7.57_415.2815m/z	Chenodeoxycholic acid	1.601787	0.020586	1.329437
12.28_426.3185m/z	Cholic acid	2.378838	0.036033	3.711219
9.21_83.0856m/z	4-Methylpentanal	1.449292	0.037237	1.020309
4.59_158.0597m/z	Pantothenic acid	1.851496	0.04241	2.01246
4.12_164.0705m/z	Tyrosine	0.451626	0.042917	3.691364
11.38_409.2914m/z	Ursocholic acid	1.419107	0.044007	1.290059
4.43_153.0234m/z	6-Mercaptopurine	0.828262	0.049989	1.163872

Adjusted *p*-value: Adjusted for multiple testing (Wilcoxon−Mann U test) with FDR control. FC: Fold change (iron deficiency vs iron administration). FC with a value >1.2 indicates a relatively higher concentration present in ID group, while a value <0.8 means a relatively lower concentration as compared with control group. VIP: variable influence on projection (VIP) values obtained from the PLS-DA model was larger than 1.0.

## Supplementary Materials

The following are available online at https://www.mdpi.com/2076-2615/10/8/1353/s1, Table S1: Primers used for gene expression analysis.
