# Supplementary material for: Effects of Iron Deficiency on Serum Metabolome, Hepatic Histology, and Function in Neonatal Piglets
Source: Animals (Basel). 2020 Aug 5;10(8):1353. doi: 10.3390/ani10081353 (PMC7460156; doi:10.3390/ani10081353)
Supplement: Supplementary file 1 [file animals-10-01353-s001.pdf]

animals

MDPI

Supplementary Table S1 Primers used for gene expression analysis.

Assay	Forward primer (5'–3')	Reverse primer (5'–3')	Accession No.
GAPDH	TGGTGAAGGTCGGA GTGAAC	TGTAGTGGAGGTCAATGAAG GG	NM_00120635 9.1
Cyp7a1	CCGCTTCTGATACCT GTGGA	GGTTTGCTCGGAGGAACTCA	NM_00100535 2.3
Cyp8p1	ACCCACTGGGCTCA AAACTC	ATGGAAAGTGAAGGGCGTGT	AH006582.2
CYP27A1	TCGAAGTTGGTGGCT TCCTC	ACTGCTGGATCAGCCTTGTC	NM_00124330 4.1
OTC	AAGCTGTCGCTGAC GAATGA	AGTCAGAGGCAGCAACTTCC	NM_00116400 2.2
AGCS	GAAGGTGACCAACG TCAAGGAT	GACTTCGTTCAGGTAGATGA AAATC	AY550053.1
AGS	CACACAGGACGAAG TCGGAA	CCTCTCGGAGTCTCTGGTCA	XM_02108636 0.1
CPS- I	AAGGCAAAGGAGAT TGGGTTC	TACTGATGGGTATTCTGCAG CC	XM_00567215 9.3
NAGS	CCTGTTCAAGAACGC CGAAC	GCGTAACGAGGCCAGATAGT	NM_00109752 0.1

GAPDH, the gene of internal reference; Cyp7a1, cholesterol 7α-hydroylase; Cyp8p1, Sterol 12α-hydroxylase; CYP27A1, sterol 27 hydroxylase; CPS- I, carbamyl phosphate synthetase I; OTC, ornithinetranscarbamoylase; NAGS, N-acetylglutamate synthase; AS, Argininosuccinate Synthetase; AL, Argininosuccinase lysozyme;.
